# Mitochondrial heteroplasmy as a transitional state in the evolution of fungicide resistance

**DOI:** 10.1128/mbio.02332-26

**Published:** 2026-10-21

**Authors:** Kathleen Schoettner, Carolin Hogl, Carola Gunkel, Cindy Vallières, Brigitte Meunier, Isabella Siepe

**Affiliations:** 1 BASF Agricultural Solutions Deutschland GmbH, Limburgerhof, Germany; 2 Université Paris-Saclay, CNRS UPR 2301, Institute de Chimie des Substances Naturelles (ICSN) 27048, Gif-sur-Yvette, France; 3 Université Paris-Saclay, CEA, CNRS, Institute for Integrative Biology of the Cell (I2BC) 27048, Gif-sur-Yvette, France; Instituto Carlos Chagas, Curitiba, Brazil

**Keywords:** cytochrome *b*, metyltetraprole, mitochondrial heteroplasmy, mutation switch, pyraclostrobin, Q_o_I resistance, *Zymoseptoria tritici*, evolution of fungicide resistance

## Abstract

Mitochondrial DNA (mtDNA) can exist in homoplasmic or heteroplasmic states, but the dynamics and stability of heteroplasmy remain poorly understood in fungal plant pathogens. Because a key fungicide target (cytochrome *b*) is encoded in the mitochondrial genome, understanding these mtDNA dynamics is crucial for predicting resistance development and evolution in agricultural pathosystems. In *Zymoseptoria tritici,* mutations G143A and Y132C in the quinone outside inhibitor-binding site of the cytochrome *b* subunit confer a high level of resistance to the fungicides pyraclostrobin and metyltetraprole, respectively. Under fungicide selection, these mutations were observed to coexist only transiently. Using experimental evolution and single-cell analysis, we demonstrate the presence of a temporarily heteroplasmic state in which both G143A and Y132C alleles coexist within individual cells at variable ratios. Allele predominance within the heteroplasmic populations shifted rapidly in response to fungicide pressure, resulting in a mitochondrial mutation switch that ultimately led to either G143A or Y132C homoplasmy. This finding provides evidence that transient heteroplasmy represents a critical intermediate phase during the transition between resistance genotypes in *Z. tritici*. To our knowledge, this is the first single-cell demonstration of mitochondrial heteroplasmy in a pathogenic fungus, offering new insights into mitochondrial genome dynamics that shape adaptive responses to fungicides.

**IMPORTANCE:**

Mitochondrial genomes in fungal pathogens are generally considered to be genetically uniform. However, our work shows that mitochondrial heteroplasmy can form a transient and unstable state that enables switching between resistance-associated haplotypes under changing selective pressures. Using single-cell analysis, we demonstrate that individual cells harbor varying proportions of resistance alleles, which can rapidly shift depending on fungicide exposure. These findings provide new insight into how mitochondrial genome dynamics contribute to microbial adaptation and highlight a previously underappreciated mechanism that may influence the emergence and development of antifungal resistance.

## INTRODUCTION

Unlike nuclear DNA, mitochondrial DNA (mtDNA) is characterized by its high copy number (1, 2). This allows two distinct genetic states: homoplasmy, in which all mtDNA molecules share the same sequence, and heteroplasmy, where multiple mtDNA variants coexist. Heteroplasmy is reported and well studied in humans (3, 4). In mammals, mtDNA mutations are linked to mitochondrial disease, aging, and cancer (5, 6). Since cells hold multiple mtDNA copies, normal mtDNA can offset abnormal variants. Phenotypes related to mtDNA mutations arise only when mutant levels exceed the mitochondrial threshold effect (4, 7).

In contrast, fungal mitochondrial genomes are generally thought to be homoplasmic. Evidence for this is seen in *S. cerevisiae*, where mutations in the cytochrome *b* gene, like those conferring resistance to complex III inhibitors, remain stable at homoplasmic levels even when selection pressure is removed (8). Similar stability has been observed in various field and laboratory fungal mutants carrying complex III inhibitor resistance even without selection pressure.

However, there are reports of heteroplasmy in fungi. In limited cases, persistent heteroplasmy has been observed. The analysis of *COX1* and *COX3* in *Thelephora ganbajun* and *ATP6* in *Cantharellus cibarius* (both basidiomycetes) indeed suggested stable heteroplasmy (9, 10). In samples of ascomycete apple powdery mildew *Podosphaera leucotricha*, both cytochrome *b* strobilurin resistance mutation G143A and its wild-type (WT) allele were maintained for years in the absence of selection pressure (11). But, in most cases, the heteroplasmy seems only transient or unstable, and the mutant allele is lost without selection pressure (reference 12 and references therein). Similarly, research on the arbuscular mycorrhizal fungus *Rhizophagus irregularis* revealed that while progeny from crossed isolates initially carry two parental mtDNA haplotypes, they revert to a single mtDNA haplotype upon germination, suggesting heteroplasmy is a transient state in this organism (13). A recent real-time imaging study of *Saccharomyces cerevisiae* has further illustrated the transient nature of mitochondrial heteroplasmy. Following the formation of a heteroplasmic zygote, parental mtDNA alleles undergo rapid segregation, reaching near-complete homoplasmy in just 24 h, facilitated by the partitioning of a small number of mtDNA molecules to the daughter cells (14).

Despite its importance, the dynamics of mtDNA have not been explored in phytopathogenic fungi, largely because the instability of the heteroplasmic state makes its monitoring difficult. However, understanding these dynamics is critical for pathogen management, as shifts in the ratio of wild-type to mutant alleles, such as those conferring cytochrome *b* inhibitor resistance, can pose significant threats depending on the growth condition.

In this study, we combined experimental evolution and single-cell analysis to monitor cytochrome *b* variants within *Zymoseptoria tritici* cells. *Z. tritici* is a major fungal pathogen that causes Septoria leaf blotch in wheat, a disease that leads to substantial crop losses worldwide (15). Fungicides targeting mitochondrial respiration, particularly complex III inhibitors known as quinone outside inhibitors (Q_o_Is, e.g., strobilurins), have been widely applied to manage this pathogen due to their strong effectiveness (16). These inhibitors bind to the Q_o_ site of complex III, which is mainly formed by the mitochondrially encoded cytochrome *b* subunit, and block the complex III catalytic activity, thus disrupting energy production and ultimately leading to fungal death.

However, the intensive use of strobilurins has driven the emergence of resistance, largely attributed to mutations within the cytochrome *b* (encoded by the *CYTB* gene). Currently, the G143A mutation represents the most problematic one, as it is widespread and confers a high degree of resistance (17).

Metyltetraprole, a more recent Q_o_I (18), is unaffected by the G143A mutation (19). Nonetheless, laboratory studies have revealed that another mutation in the cytochrome *b*, Y132C, confers resistance to this fungicide (20).

Since G143A is now prevalent in most of the *Z. tritici* isolates, an important question arose: Can both mutations, Y132C and G143A, coexist within the same population? Addressing this question is essential for developing sustainable fungicide management strategies.

A previous study found that a stable *Z. tritici* double mutant (Y132C + G143A) could not be selected, likely due to functional incompatibility (20). In this paper, we confirmed this and detected mutants with varying levels of both mutations, demonstrating mitochondrial heteroplasmy. Our findings show heteroplasmy is temporary and provide the first direct molecular evidence of its role in fungicide-dependent mutation switching in pathogenic fungi. Using the yeast model *Saccharomyces cerevisiae*, we further demonstrated that the combination of Y132C and G143A resulted in a severe respiratory growth defect.

## MATERIALS AND METHODS

### *Z. tritici* isolate, growth and compound treatment

The previously described *Z. tritic*i isolate *CYTB*_Y132C#3 (20), here called Y132C, was used in this study.

*Z. tritici* cells were cultivated on YBG (10 g/L yeast extract, 10 g/L bacto peptone, and 20 g/L 99% glycerol), where the fungus grows as yeast-like cells, with or without fungicide(s). For direct evolution experiments, *Z. tritici* cells were grown in 30 mL YBG liquid medium supplemented with the fungicide(s) in 125-mL flasks, at 23°C, with agitation at 150 rpm.

Growth was monitored using the Cell Growth Quantifier (CGQ) from Scientific Bioprocessing (SBI). The CGQ enables non-invasive, real-time monitoring of microbial growth by measuring light scattered from cell suspensions in Erlenmeyer flasks (21). Increased cell density results in greater light scattering, providing immediate insights into biomass changes. We previously showed that the CGQ measurements were consistent with the classical optical density measurements at 600 nm (20).

The directed evolution experiment was done as described in reference 20. In short, the Y132C mutant examined in this study was first cultured in the presence of 0.02 µM pyraclostrobin, a concentration that caused approximately 50% growth inhibition until the culture reached a plateau as determined by CGQ monitoring. An aliquot of the culture was then taken to inoculate a fresh culture. A new treatment cycle was initiated and continued until the culture reached a plateau. Eight cycles of growth were performed. The inhibitor concentration was doubled with each cycle.

At the end of the eighth cycle, an aliquot of the culture was used to start a new treatment experiment using both pyraclostrobin and metyltetraprole. The culture was split into two parallel flasks representing two selection regimes (#1 and #2). In selection regime #1, the starting concentrations were 1.28 µM pyraclostrobin and 0.125 µM metyltetraprole, with the metyltetraprole concentration reduced to 0.064 µM from cycle 12 onward to allow better growth. In selection regime #2, the parallel culture was exposed to higher concentrations of both fungicides, namely 2.56 µM pyraclostrobin and 0.25 µM metyltetraprole. Aliquots were collected at the end of each cycle for sequencing or qPCR analysis. Cell suspensions were also streaked onto YBG solid medium, with or without fungicides. Colonies were allowed to form and further propagated with or without fungicides.

Initially, fungicide concentrations were chosen with the aim of generating comparable selection pressures on the Y132C and G143A alleles. However, as qPCR analyses became available and provided insight into the evolving allele frequencies, we found that equivalent selective pressures cannot be readily defined in heteroplasmic populations. Consequently, concentrations within selection regime #1 were adjusted to sustain growth and continue the selection experiment.

### Monitoring the frequencies of *CYTB* mutations Y132C and G143A in *Z. tritici* populations and single cells

#### DNA extraction

Samples of each cell population were harvested. Genomic DNA was extracted from pelleted cells using the DNeasy PowerSoil Pro Kit from Qiagen following the manufacturer’s protocol.

#### qPCR assay

A total of 0.5–1 ng genomic DNA was added together with 0.5 µM of each primer (listed in Table 1), 0.083 µM of TaqMan probe (FAM-probe), and 12.5 µL of 2x Takyon No ROX Probe MasterMix, in a 20 µL reaction. Thermal cycling was performed using a two-step protocol, with an initial denaturation at 95°C for 5 min, followed by 40 cycles of 95°C for 10 s and 60°C for 45 s. Reactions were carried out on the CFX Opus 96 Real-Time PCR System (Bio-Rad), and data were analyzed using the CFX Maestro Software (Bio-Rad). All reactions were set up in duplicate. Relative quantities were calculated using the 2^−[Δ][Δ]Ct^ method with the wild-type reference strain IPO323 and single mutants G143A and Y132C as controls.

**TABLE 1:** Primers used in this study

Name	Type		Sequence (5′–3′)
	qPCR primers	Used for the detection of	
Web2062	Reverse	Y132, Y132C, G143, G143A	CCCTAGAACATTAACATGAACAATCG
Web2060	Forward	Y132	CTCATAAAGACATTTGACCATAAGGTAATACCT
Web2061	Forward	Y132C	CTCATAAAGACATTTGACCATAAGGTAATACTC
Web2064	Forward	G143	CAATAAGTTAGTTATAACTGTTGCCC
Web2065	Forward	G143A	GCACTCAATAAGTTAGTTATAACTGTTGCAG

	Fluorescent reporter for qPCR		
Web2063	FAM-probe	CCCTAAGAATGCGGTTGCCATCATCA	

	Primers for sequencing and amplification		
Web1973	Forward	Sequencing	TGCCTATTGACATATACCTGACTAG
Web1974	Reverse	Sequencing	ACCATCACTATGTATCATCACTGAT
Web2101	Forward	Sequencing and amplification	TCTTCACTCTAATACTGCTTCAGCT
Web2104	Reverse	Sequencing and amplification	TCTACCTAAATCGGTAATTGGCA
Web2099	Forward	Amplification	AATGGTTCTACTGGCCCTGC
Web2100	Reverse	Amplification	GCAGCTTCGTCCACAATAGC

#### Single-cell preparation for digital PCR

A cell suspension of *Z. tritici* was prepared in YBG medium. The cell concentration was determined using a Neubauer counting chamber. Serial dilution steps were performed to adjust the final spore concentration to 1 spore per 10 µL. Subsequently, 10 µL of the diluted suspension was distributed into the wells of a half-area microtiter plate. Each well was examined under an inverted microscope, and only those containing exactly one cell were selected for further experiments.

Single cells were boiled at 98°C for 10 min, followed by PCR using primers Web2101 and Web2104 (Table 1) with Phusion Flash High-Fidelity PCR Master Mix (50 cycles, 50 µL). PCR products were purified (QIAquick 96 PCR Purification Kit), diluted 1:10⁶, and analyzed by the digital PCR (dPCR) method.

#### Digital PCR

A total of 0.4 ng of the PCR products was used in a 12 µL reaction containing 0.2 U/µL of AluI enzyme, 20× ddPCR mutation assay, including primers, wild-type and mutant probes (customized from Bio-Rad), and 4× QIAcuity Probe PCR Kit. PCR was performed using 96-well nanoplates with 8,500 partitions per well (nanoplate 8.5K) on the QIAcuity One 5plex System, and data were analyzed with the QIAcuity Suite Software. Assay controls, partition quality criteria, confidence intervals, and independent validation runs were used (22). Technical replicate wells were not performed, as QIAcuity dPCR does not require replicate reactions according to reference 22.

#### Sequencing and subcloning

The *CYTB* gene was amplified by PCR using primers Web2099 and Web2100 and subsequently sequenced using primers Web1973 and Web1974 (Table 1).

For subcloning, a *CYTB* gene fragment was PCR-amplified with primers Web2101 and Web2104, purified, cloned with the Zero Blunt TOPO kit, and transformed into *E. coli* One Shot cells. The sequencing was done using the standard M13 forward and M13 reverse primers.

### *Saccharomyces cerevisiae* culture media and strains

The following media were used for the growth of yeast: YPD (1% yeast extract, 2% peptone, 3% glucose, 2% agar), YPG (1% yeast extract, 2% peptone, 3% glycerol, 2% agar), and YPEth (1% yeast extract, 2% peptone, 2% ethanol).

The control strain (WT) and the cytochrome *b* mutants (G143A, Y132C, and Y132C + G143A) were diploids (Mat α ura3 his3 leu2 ade2 trp1/Mat a leu1 kar1-1) with isogenic intronless mitochondrial DNA, with the exception of the point mutations in the cytochrome *b* gene *CYTB*, and were homoplasmic.

The strains were already available in our lab (23), with the exception of the double mutant Y132C + G143A that was generated in this study.

The generation of the mutants was performed by the technique of biolistic transformation as previously described (24, 25). Please see Supplemental material for a detailed method description.

## RESULTS

### Fluctuation in the frequencies of cytochrome *b* Y132C and G143A mutations in *Z. tritici* populations in response to fungicide exposure

In order to address the question of coexistence of two relevant cytochrome *b* mutations, we investigated the effect of exposure to pyraclostrobin (a strobilurin fungicide) and metyltetraprole on the frequencies of Y132C (resistance to metyltetraprole) and G143A (resistance to pyraclostrobin) mutations in *Z. tritici*.

In a previous study, two independent Y132C mutant strains were subjected to three evolution experiments in which they were cultured for 8 or 10 growth cycles, depending on the treatment regime, in the presence of increasing pyraclostrobin concentrations ranging from 0.02 µM to 5.12 µM (20). Across all three experiments, a reproducible shift from Y132C to G143A-containing mitochondrial genomes was observed. Notably, this transition consistently began at pyraclostrobin concentrations of 1.28 µM or higher.

**Fig 1:**
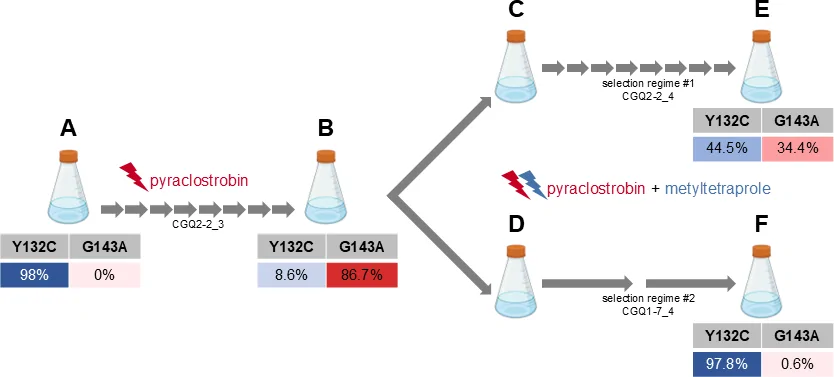
Effect of pyraclostrobin and metyltetraprole treatment on the dynamics of cytochrome *b* mutations Y132C and G143A during experimental evolution. Mutant Y132C (step A) was cultivated for eight cycles with increasing concentrations of pyraclostrobin (step B) (20). Two aliquots (steps C and D) thereof were further treated with pyraclostrobin and metyltetraprole. Aliquot C was exposed to 1.28 µM pyraclostrobin and 0.125 µM metyltetraprole for three cycles followed by five cycles at 1.28 µM pyraclostrobin and 0.064 µM metyltetraprole (selection regime #1). Aliquot D was exposed to higher concentrations (2.56 µM pyraclostrobin and 0.25 µM metyltetraprole) for two cycles (selection regime #2). At the end of each respective experiment (selection regimes #1 and #2), samples were analyzed by qPCR (steps E and F).

For this study, we selected one representative population (CGQ2-2_3, named selection regime #5 in reference 20) of these three cultures for detailed characterization. In that culture, at the end of eight cycles of pyraclostrobin treatment, qPCR analysis revealed a drastic shift in the genetic profile of the population, where the frequency of the Y132C mutation plummeted to 8.6%, while the G143A mutation rapidly emerged and achieved strong dominance, reaching 86.7% of the population (Fig. 1, step B).

This mixed Y132C/G143A population was subsequently split into parallel cultures (Fig. 1, steps C and D) and further propagated under different selection pressures with both metyltetraprole and pyraclostrobin to investigate the dynamics of the mitochondrial haplotype switch under different QoI selection regimes. One culture was exposed to 1.28 µM pyraclostrobin and 0.125 µM metyltetraprole for three selection cycles, which was followed by five cycles at 1.28 µM pyraclostrobin and 0.064 µM metyltetraprole (selection regime #1). The second culture was exposed to 2.56 µM pyraclostrobin and 0.25 µM metyltetraprole for two cycles (selection regime #2). At the end of each cycle, qPCR analyses were performed to monitor the changes in mutation frequencies.

**Fig 2:**
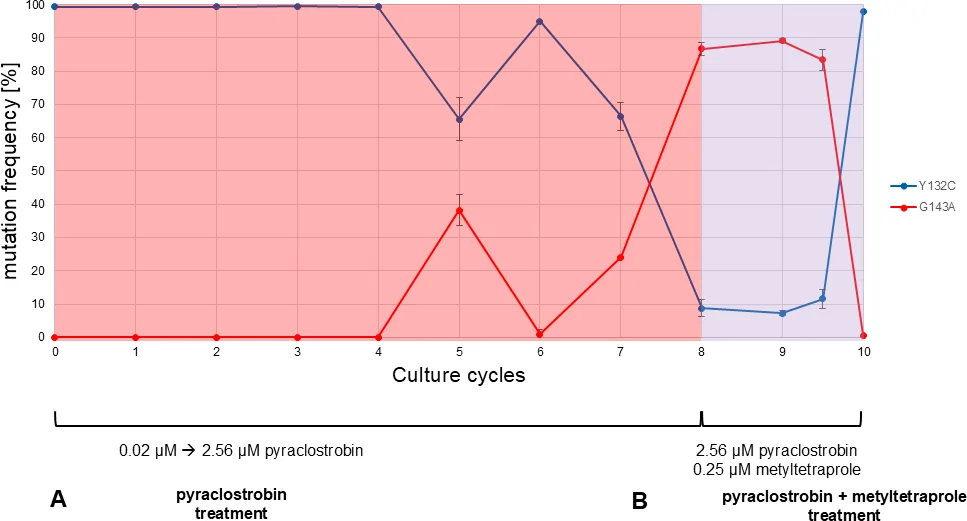
Effect of pyraclostrobin and metyltetraprole treatment (selection regime #2) on the frequencies of cytochrome *b* mutations Y132C and G143A (20). Mutant Y132C was cultivated for eight cycles with increasing concentrations of pyraclostrobin from 0.02 µM to 2.56 µM (**A**, red background, see also reference 20). An aliquot thereof was further treated with both pyraclostrobin (2.56 µM) and metyltetraprole (0.25 µM) for an additional two cycles (**B**, violet background). At the end of each cycle, qPCR analyses were performed. The percentages of mutations Y132C (blue) and G143A (red) measured by qPCR are presented.

The culture maintained at the higher fungicide concentrations (selection regime #2) rapidly underwent a complete switch (Fig. 1, step F, and Fig. 2).

While G143A initially dominated the population after cycle 8, this state proved transient when exposing the culture to high concentrations of both pyraclostrobin and metyltetraprole. A second switch occurred, and Y132C genomes again became predominant (Fig. 2; Table S1). Interestingly, the culture subsequently stopped growing, yet viability controls confirmed the persistence of living cells (Fig. S1).

**Fig 3:**
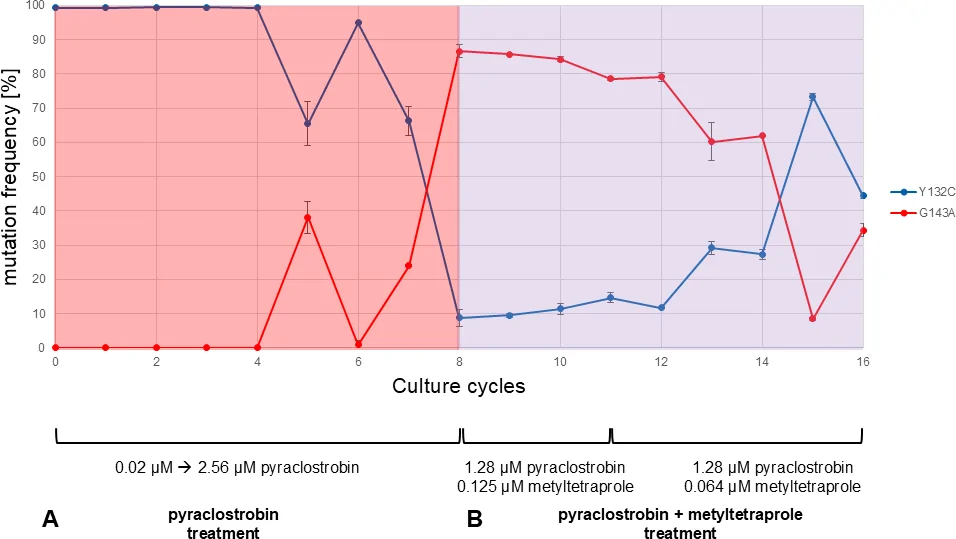
Effect of pyraclostrobin and metyltetraprole treatment (selection regime #1) on the frequencies of cytochrome *b* mutations Y132C and G143A. Mutant Y132C was cultivated for eight cycles with increasing concentrations of pyraclostrobin from 0.02 µM to 2.56 µM (**A**, red background;, see also reference 20). An aliquot thereof was further treated with both pyraclostrobin (1.28 µM) and metyltetraprole (0.125 µM for cycles 8–11; 0.064 µM for cycles 12–16) (**B**, violet background). At the end of each cycle, qPCR analyses were performed. The percentages of mutations Y132C (blue) and G143A (red) measured by qPCR are presented.

In contrast, the culture exposed to the lower fungicide concentrations (selection regime #1) followed a slightly different trajectory (Fig. 3; Table S2; Fig. S2). Although both mutations fluctuated over time, neither mutation reached fixation during the experiment. By the end of cycle 16, the population consisted of a mixed genotype distribution, with 44.5% of cells carrying the Y132C mutation and 34.4% carrying G143A. These results indicate that the lower selection pressure allowed the unstable and varying coexistence of both resistance variants over an extended period, whereas complete switching was observed more rapidly under the higher fungicide concentrations.

To determine whether both mutations coexisted within the same allele, an aliquot of the population collected at the end of cycle 16 was subjected to DNA extraction and 192 *CYTB* PCR amplicons were sequenced. Y132C and G143A single-mutant alleles were observed, as well as Y132C + G143A double mutant alleles, but at low frequency (Table 2; Fig. S3).

**TABLE 2:** Summary of the analysis of 192 *CYTB* PCR-amplified gene fragments from the cycle 16 cell population (selection regime #1)

	Number of amplicons	Percentage
Total	192	
Y132C	80	41.7
G143A	106	55.2
Y132C + G143A	5	2.6
Wild type (Y132 + G143)	1	0.5

Sequencing and analysis of the *CYTB* PCR fragments from these numerous individual clones confirmed the qPCR-based estimates of mutation frequencies.

**Fig 4:**
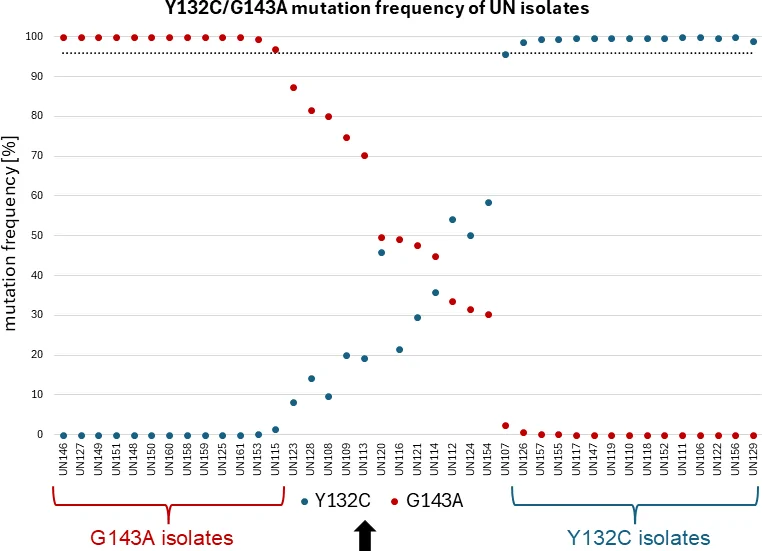
Y132C and G143A mutation frequencies of UN clones from selection regime #1 analyzed by qPCR. The UN clones are sorted by mutation frequency. UN113 is marked by an arrow.

To examine the dynamics of mutation-frequency shifts and to try to select pure double mutants, cells from the culture at the end of cycle 16 of selection regime #1 were streaked onto YBG to obtain single colonies. After incubation, individual colonies were picked and further cultivated without fungicide treatment; these isolates were named UN (untreated) clones. A total of 40 UN clones were selected and analyzed by qPCR. Mutation frequencies above 96% were indicative of a “pure” strain (either G143A or Y132C). Among the 40 UN clones, 13 (32.5%) were classified as G143A mutants, 14 (35%) as Y132C mutants, and 13 (32.5%) contained both alleles at varying proportions (Fig. 4; Table S3; Fig. S5). Sequencing of 10 UN clones confirmed the qPCR results and revealed double peaks at codons 132 and 143 (Fig. S4). Neither clone showed both mutations exceeding 96%, indicating that there was no single clone with an allele carrying both the G143A and Y132C mutations together at a homoplasmic level. This result points to a possible incompatibility between these two mutations.

In a similar approach, cells collected at the end of cycle 10 under selection regime #2 were plated on YBG agar containing 2.56 µM pyraclostrobin and 0.25 µM metyltetraprole. The resulting clones, designated MP clones, were analyzed by qPCR (Table S4). Among these, MP393 contained both Y132C (82.17%) and G143A (10.54%) mutations (see also Fig. S5). Sequencing of 66 *CYTB* PCR amplicons identified Y132C and G143A single-mutant alleles, as well as one double-mutant allele (Table 3; Fig. S6). Overall, the mutation frequencies determined by PCR amplicon sequencing were consistent with the qPCR results.

**TABLE 3:** Summary of the analysis of 66 *CYTB* PCR-amplified gene fragments from the MP393

	Number of amplicons	Percentage
Total	66	
Y132C	62	93.9
G143A	3	4.5
Y132C + G143A	1	1.5
Wild type (Y132 + G143)	0	0

**Fig 5:**
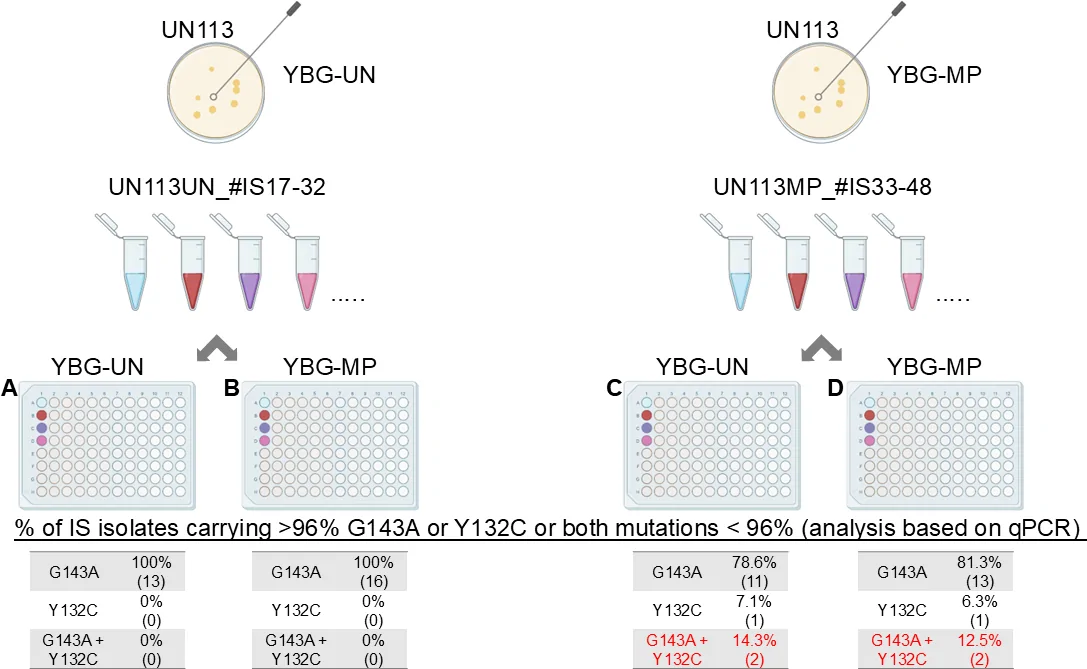
Analysis of cells derived from clone UN113 after growth in the presence or absence of fungicides. UN113 was streaked on YBG without fungicide (YBG-UN) and on YBG supplemented with 0.064 µM metyltetraprole and 0.128 µM pyraclostrobin (YBG-MP). Cell suspensions from individually picked UN113 colonies (UN113UN_#IS17-32 and UN113MP_#IS33-48) were used to inoculate YBG without fungicide (YBG-UN; **A and C**) and YBG with fungicides (YBG-MP; **B and D**). After growth, DNA was extracted for the cell populations and qPCR analyses were performed. Also, the cell suspension obtained after the colony was resuspended was analyzed. The numbers of isolates carrying the G143A mutation, Y132C mutation, or both are shown. Figure was created with BioRender.com.

We further extended our analysis by examining how mutation frequencies fluctuated under various growth conditions in the descendants of clone UN113, which showed 19% Y132C and 70% G143A based on qPCR data (Fig. 4; Table S3). UN113 cells were streaked on both YBG medium (YBG-UN) and YBG medium supplemented with metyltetraprole (0.064 µM) and pyraclostrobin (0.128 µM) (YBG-MP). After growth, 16 colonies were selected from YBG (UN113UN_#IS17 to 32) and 16 from YBG +MP (UN113MP_#IS33 to 48) (Fig. 5). Each colony was resuspended in YBG, and the cell suspensions were split to inoculate both YBG and YBG-MP liquid media. In this way, cells from single, presumably clonal colonies were exposed to different treatments, facilitating comparison. After 4–6 days of growth, DNA extraction followed by qPCR analysis was performed for each cell population. All the cell populations derived from colonies grown on fungicide-free solid medium (YBG-UN) (Fig. 5A and B) carried only the G143A mutation (level of mutated allele >96%). The Y132C allele was most likely lost during the formation of the colonies and remained absent even after growth in liquid YBG-MP (the incubation period in YBG-MP was likely insufficient to allow the reemergence of the Y132C mutation).

On the other hand, when single colonies isolated from YBG-MP solid medium were further propagated, both pure G143A and pure Y132C mutants (level of mutated allele >96%) could be recovered (Fig. 5C and D). Additionally, four isolates displayed both mutations at varying levels below 96%; they were named UN113MP#IS45UN, UN113MP#IS45MP, UN113MP#IS46UN, and UN113MP#IS46MP (Table 4).

**TABLE 4:** Levels of Y132C and G143A mutations in isolates from colonies derived from UN113^*a*^

Colonies	Propagation in liquid media	Isolate name	Y132C (%)	G143A (%)
#IS45	YBG-UN	UN113MP#IS45UN	5.4	85.3
	YBG-MP	UN113MP#IS45MP	30.8	59.6
#IS46	YBG-UN	UN113MP#IS46UN	3.8	92.8
	YBG-MP	UN113MP#IS46MP	60.8	22.4

^*a*^ Isolates originated from UN113 colonies grown on YBG-MP. The cell suspension of the picked colonies was further propagated in YBG-UN and YBG-MP independently. qPCR analyses of the isolates were performed to determine the mutation frequencies.

Y132C and G143A frequencies in these isolates varied depending on the medium in which they were propagated (Table 4). When grown in YBG-UN, the mutation profile tended to shift strongly toward G143A (85.3% and 92.8%). In contrast, cultures grown in YBG-MP displayed a more balanced distribution between the two mutations; UN113-MP#IS45MP showed 30.8% Y132C and 59.6% G143A, and UN113-MP#IS46MP exhibited 60.8% Y132C and 22.4% G143A.

Again, a pure double mutant (both mutations at >96%) was not detected, which supports an incompatibility between G143A and Y132C. The loss of the Y132C allele during prolonged periods without selection pressure suggests that this mutation may impose a fitness cost on the fungal population that could thus be further aggravated by the presence of G143A.

Our results also indicate that sequential or combined exposure to pyraclostrobin and metyltetraprole in *Z. tritici* generates complex shifts in resistance allele frequencies. The detection of both mutations at varying frequencies within single isolates, even after rounds of subculturing, strongly suggests the occurrence of mitochondrial heteroplasmy, potentially representing a transient intermediate state before the population resolves into a homoplasmic genotype.

### Heteroplasmy observed in *Z. tritici* at single-cell level

To further analyze the shift in resistance allele frequency and monitor heteroplasmy at the cell level, we quantified the mutations in single cells. The amplified *CYTB* PCR products from 30 individual single cells derived from UN113-MP#IS46MP were analyzed through digital PCR (dPCR) (Fig. S7).

**Fig 6:**
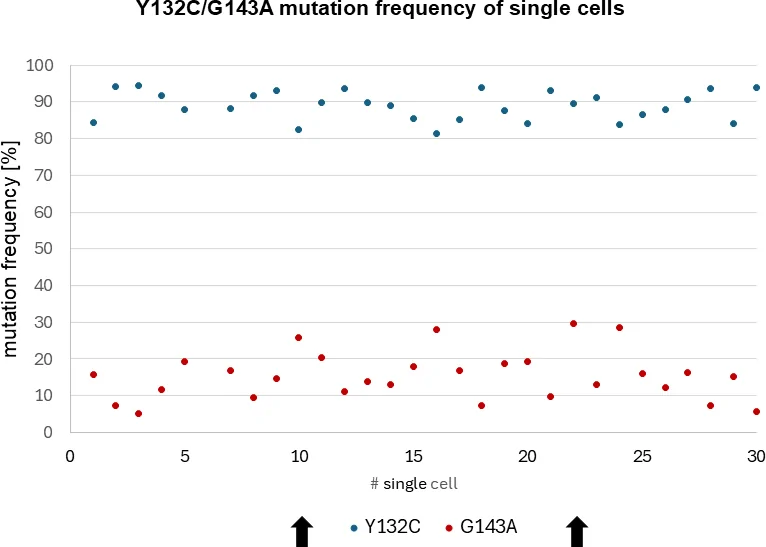
Y132C and G143A mutation frequencies of single cells derived from UN113-MP#IS46MP analyzed by dPCR. UN113MP#IS46 single cells 10 and 22 are marked by arrows.

**Fig 7:**
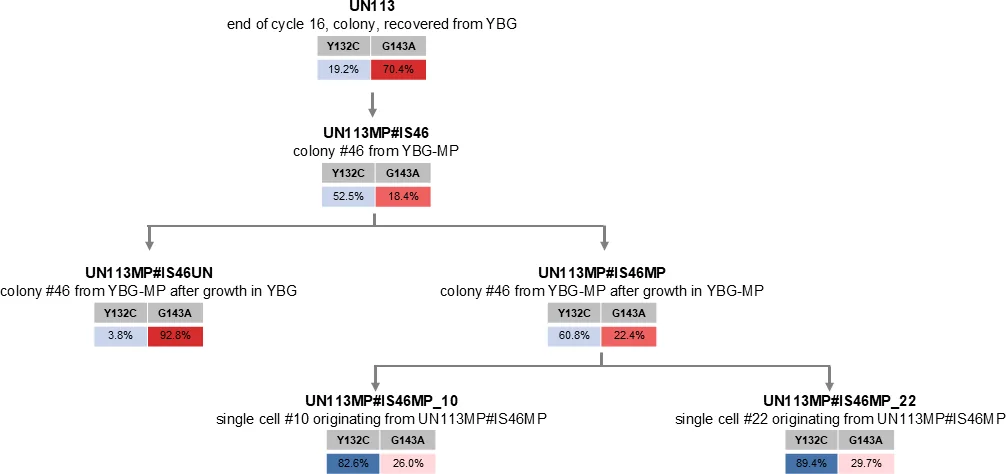
Examples of varying mutation frequencies of UN113 throughout the experiments. Initial analysis of UN113 and recovered colonies and single cells thereof. The cell suspension of UN113MP#IS46 was directly analyzed without further growth, while UN113MP#46MP was further propagated in YBG-MP. Single cells #10 and #22 of UN113#IS46MP were taken from frozen glycerol stock and analyzed later. qPCR and dPCR (single-cell) data emphasize the unstable heteroplasmy (MP, treatment with metyltetraprole and pyraclostrobin; UN, untreated).

As presented in Fig. 6 and Table S6, both mutations were detected in varying proportions. Their ratio differed among single cells, even though they were derived from the same isolate. The mtDNA populations seemed to have shifted compared to the first measurement with the cell suspensions of UN113MP#IS46MP (Fig. 7). In all the single cells, Y132C frequency indeed increased to above 80% compared to 60.8% measured in UN113MP#IS46MP (see Table 4), and G143A frequency varied between 5% and 30%. For a single cell, namely UN113MP#IS46MP_22 (Table S6), the dPCR results were validated by sequencing of 44 *CYTB* PCR products as well as deep sequencing (data not shown).

The frequency shift between UN113MP#IS46MP and its individual cells may stem from PCR inaccuracies using single cells or unequal selection pressures from pyraclostrobin and metyltetraprole, favoring Y132C. A similar mutation shift was noted from UN113 to UN113MP#46 after exposure to both fungicides. Overall, these results confirm single-cell heteroplasmy and highlight its pronounced instability.

Analysis of single cells derived from UN113-MP#IS45MP was also performed and showed an even more severe shift toward homoplasmy for Y132C and a decrease in G143A frequency (Table S5), confirming the instability of the heteroplasmic state.

Throughout the experiments, UN113 and its descendants demonstrated different mutation frequencies based on age and growth conditions. A selected compilation is presented in Fig. 7.

As already mentioned, cells from the end of cycle 10 of selection regime #2 were also analyzed. After growth on YBG agar supplemented with 2.56 µM pyraclostrobin and 0.25 µM metyltetraprole, the isolates were analyzed by qPCR. Isolate MP393 was found to contain both the G143A (82.17%) and Y132C (10.54%) mutations (Table S4).

In an analogous manner to single-cell analysis performed for the UN113 clones, MP393 was then analyzed further by dPCR to determine whether both mitochondrial mutations were present within individual cells (Table S7). Among the single cells analyzed, MP393_4 showed signals for both Y132C (96.06%) and G143A (2.05%), indicating the presence of both mutations. This result was further confirmed by sequencing of cloned *CYTB* PCR fragments, which detected both mutation types among the sequenced fragments (Table 5; Fig. S8).

**TABLE 5:** Summary of the analysis of 94 *CYTB* PCR-amplified gene fragments from single-cell MP393_4

	Number of amplicons	Percentage
Total	96	
Y132C	94	97.9
G143A	2	2.0
Y132C + G143A	0	0
Wild type (Y132 + G143)	0	0

### Interpretation and summary of key findings focusing on selection regime #1

**Fig 8:**
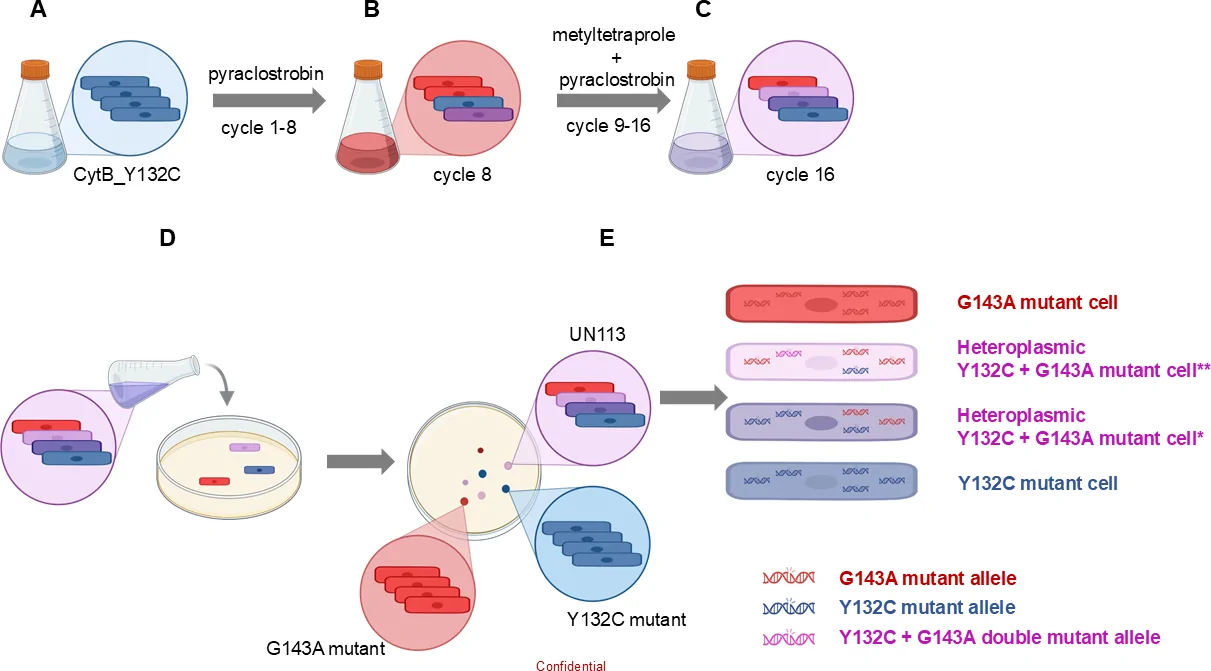
Model of mutation switching involving the heteroplasmic “transition” stage. The experiment started with the Y132C mutant (**A**). Upon treatment with increasing concentrations of pyraclostrobin for eight cycles, the cell populations contained different types of mutants (**B**). G143A and Y132C could be recovered, as well as mutants carrying both mutations to varying degrees. When plated on solid media (**C**), each single cell started forming a colony (**D**). During multiplication, individual cells originating from the same single cell could display varying mutation levels. The single cells are either homoplasmic or heteroplasmic. Heteroplasmic cells carry both the Y132C and G143A mutant alleles (**E**, *). In rare cases, the Y132C + G143A double mutant allele is present (**E**, **). Figure was created with BioRender.com.

After eight cycles of pyraclostrobin treatment, analysis of the initial Y132C cell culture revealed the presence of both Y132C and G143A mutations, with G143A becoming the predominant mutation while the frequency of Y132C decreased (Fig. 8A and B). Sanger sequencing of all recovered isolates showed either the Y132C or G143A mutation in the mitochondrially encoded *CYTB* gene. Following combined unbalanced treatment with pyraclostrobin and metyltetraprole, the proportion of both mutations shifted toward Y132C (Fig. 8C). These cells were streaked, and the resulting cell colonies were analyzed by qPCR, revealing pure Y132C and pure G143A isolates, as well as colonies with varying proportions of both mutations, for example, UN113 (Fig. 8D and E). After an additional round of cell separation, isolates with different percentages of each mutation persisted.

Single-cell dPCR detected heterogeneity within isolates, with individual cells from the same isolate displaying varying mutation levels. Further subcloning of *CYTB* from single cells confirmed the presence of G143A, Y132C, and the G143A + Y132C double mutant alleles within a single cell (Fig. 8E).

### Respiratory growth defect caused by the double mutation Y132C + G143A in *Saccharomyces cerevisiae*

As shown above, in *Z. tritici*, amplicons with both Y132C and G143A were only detected at low levels, and no mutant carrying both mutations at homoplasmic levels could be obtained, suggesting that the two mutations could not coexist without a significant penalty.

To evaluate the impact of the double mutation Y132C + G143A, the mutations were introduced into *S. cerevisiae* using the biolistic transformation technique. So far, mitochondrial transformation is not available for *Z. tritici*. Only *S. cerevisiae* and the green alga *Chlamydomonas reinhardtii* are amenable to mitochondrial transformation (26), which allows the introduction of designed mutations into mitochondrially encoded genes, such as *CYTB*.

**Fig 9:**
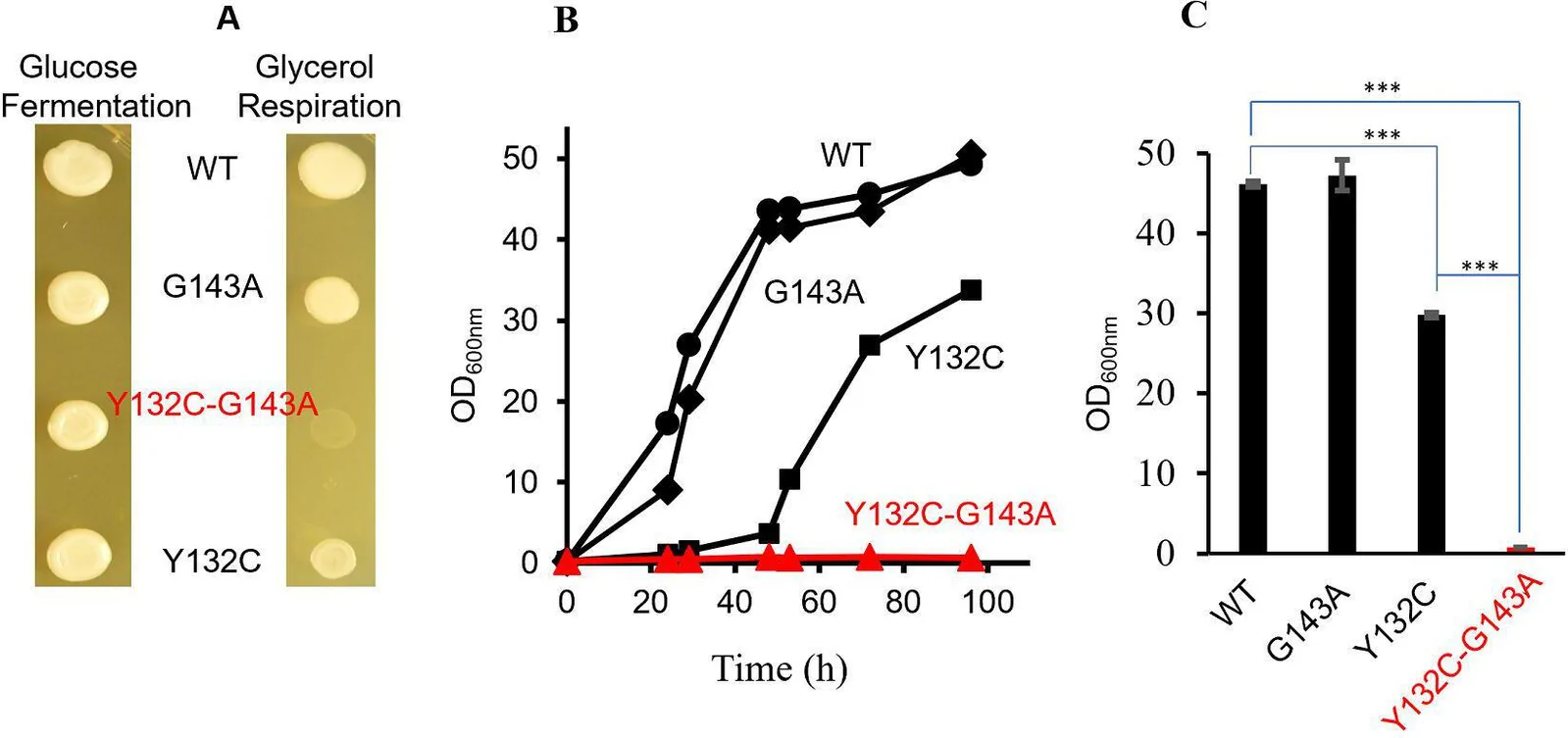
Yeast. Respiratory growth competence of the cytochrome b mutants. (**A**) WT and mutant strains were cultured on YPD overnight. Cell suspensions were then spotted on glucose (YPD) and glycerol (YPG) plates and incubated for 1 day (YPD) or 3 days (YPG). (**B and C**) Freshly grown cells on YPD were inoculated into 10 mL (**B**) or 1 mL (**C**) YPEth at an initial OD_600_ of 0.2. (**B**). The OD_600_ was monitored over a 4-day time course. (**C**) The OD_600_ was measured after 3 days of culture. The experiments were repeated twice, and the data were averaged. The error bars represent standard deviation. ***, *P* < 0.0005 according to Student’s *t*-test, two-tailed.

The growth of the double mutant Y132C + G143A was compared to that of the WT and the single mutants carrying either G143A or Y132C (Fig. 9). All four strains were isogenic (nuclear and mitochondrial genomes), differing only by the point mutations in cytochrome *b* present at homoplasmic levels. While they all grew well on fermentable carbon sources (glucose, Fig. 9A), pronounced growth differences were observed on non-fermentable carbon sources (glycerol and ethanol). G143A had no detectable effect on respiratory growth, whereas Y132C significantly impaired growth. The decreased growth of mutant Y132C was already observed on plates (Fig. 9A), but more clearly marked in liquid culture assays. In the time-course assay, Y132C exhibited slower growth compared to WT and G143A (Fig. 9B). After a 3-day culture, Y132C reached approximately 50% of the OD_600_ measured for the WT and G143A strains (Fig. 9C).

The double mutant Y132C + G143A failed to grow entirely. After a long incubation on YPG (>2 weeks), very weak growth could be observed (not shown).

Amino acids Y132 and G143 are located in the vicinity of the catalytic site Q_o_ of complex III. Replacement of G143 by an alanine has little effect on complex III activity, as previously observed (23), while Y132C perturbs complex III activity, leading to respiratory growth impairment (27). The Y132C mutation may have a similarly deleterious effect in *Z. tritici*, as Y132C mutants formed colonies of smaller size than G143A mutants or parent cells with WT cytochrome *b* (20). This interpretation is also consistent with the observed loss of the Y132C allele during extended periods in the absence of selective pressure.

The combination of Y132C with G143A seems to enhance the deleterious effect of the mutation, resulting in a complete respiratory growth defect in *S. cerevisiae*. A similar effect may occur in *Z. tritici* complex III, which could explain why no homoplasmic double mutant was selected.

## DISCUSSION

In nature, mitochondrial genomes in eukaryotes are generally considered homoplasmic, and heteroplasmy is thought to be limited both in space and time (10). However, there is limited evidence for both persistent and transient existence of heteroplasmy in different fungal species (10, 11, 28, 29).

So far, studying heteroplasmic dynamics has remained challenging regardless of the organism because it has not been possible to infer the quantity of mtDNA variants within living cells over time (14).

In this study, we demonstrate transient mitochondrial heteroplasmy in single cells of *Z. tritici*. In order to evaluate the resistance risk of Y132C + G143A double mutants and to judge their fitness and potential field relevance, we aimed to obtain homoplasmic double mutants Y132C + G143A in *Z. tritici*. Several attempts were unsuccessful, indicating that the mutations were mutually exclusive. This was further confirmed by studies using a homoplasmic Y132C + G143A *S*. *cerevisiae* mutant, in which the double mutation caused a severe defect in respiratory growth. Most likely, the combined Y132C + G143A mutations would have a similar impact in *Z. tritici*, prohibiting the selection of homoplasmic double mutants.

However, heteroplasmic strains could be obtained, although unstable. Heteroplasmy was observed in single cells, and cells derived from the same clonal colony exhibited different ratios of Y132C and G143A, which underscores the dynamic nature of the heteroplasmic state.

Depending on the growth conditions, the frequency of the G143A or Y132C allele shifted, indicating potential cellular incompatibility between these mutations. In the absence of fungicide selection, Y132C tended to be lost, as the mutation was associated with a fitness cost, as mentioned above. The G143A allele appeared more stable.

Under our experimental conditions, applying unbalanced concentrations of pyraclostrobin and metyltetraprole resulted in a notable trend toward an increased frequency of the Y132C allele. Efforts to determine fungicide mixture concentrations that would apply similar selection pressure to both mutations (Y132C and G143A) were unsuccessful due to underlying unstable heteroplasmy, which resulted in constantly changing mutation frequencies.

G143A + Y132C double mutant alleles were rarely detected throughout the experiments; they would be counter-selected because of the strong deleterious impact on respiratory function. Thus, Y132C and G143A would be unable to stably coexist. The presence of both mutations might represent a molecular switching event from one mutation to the other. The ratio of the two mutations fluctuates depending on treatment or growth conditions and eventually transitions to a homoplasmic state. In addition, each single cell seems to possess a different ratio of mutations even if they are derived from the same clonal population and similarly treated. The presumably random distribution of mutant alleles in each cell might allow them to respond differently to the change in growth conditions. The instability and heterogeneity of the heteroplasmic state might allow the pathogen to adapt rapidly to fungicide treatment.

Some similarity could be found in studies with the malaria parasite. In *Plasmodium falciparum*, the cytochrome *b* mutation Y268S confers high levels of resistance to the complex III inhibitor atovaquone (the mutation was reported in several cases of atovaquone treatment failure), but also causes a complex III defect (30, 31). Y278S seems to arise *de novo* in the population of parasites in infected patients, first at a low frequency that could significantly increase after several rounds of parasite replication under selective pressure. Computer simulation showed that each infected patient could carry parasites with Y278S at varied low frequencies, with parasites harboring varied proportions of the mutant allele in their mitochondrial genome. This results in heterogeneity between infected patients. In some patients, the higher level of Y278S leads to failure to eliminate the resistant parasites despite atovaquone; the resistant parasites would then proliferate and cause treatment failure (32).

In summary, our work has shown that when *Z. tritici* is exposed to two different complex III fungicides, both *CYTB* resistance mutations, G143A and Y132C, can only temporarily coexist within individual fungal cells. This heteroplasmic state is not stable. The presence of both alleles in a single cell likely indicates the transition from one homoplasmic state to another. The ratio of the two alleles seems to fluctuate depending on treatment or growth conditions. Interestingly, the changes in allele frequency differ from single cell to single cell, even if they originate from the same colony and were treated identically. Over time, one mutation usually becomes dominant, highlighting heteroplasmy as a transitional phase.

In addition, the presence of double mutant alleles appears to result in a significant fitness disadvantage, as no persistent homoplasmic double mutants have been isolated. We assume that this double mutant allele resembles the molecular transition of the mutation switch from G143A to Y132C and vice versa. Moreover, the inheritance of heteroplasmy does not occur uniformly. Each individual cell exhibits a unique level of heteroplasmy.

## Data Availability

All data generated or analyzed during this study are included in this published article and its supplemental information files.

## SUPPLEMENTAL MATERIAL

Supplemental Figures mbio.02332-26-s0001.docx Figures S1 to S8.

Supplemental Tables mbio.02332-26-s0002.xlsx Tables S1 to S7.

Supplemental material mbio.02332-26-s0003.docx Supplemental methods.

ASM does not own the copyrights to Supplemental Material that may be linked to, or accessed through, an article. The authors have granted ASM a non-exclusive, world-wide license to publish the Supplemental Material files. Please contact the corresponding author directly for reuse.
