# Supplementary material for: Mitochondrial heteroplasmy as a transitional state in the evolution of fungicide resistance
Source: mBio. 2026 Oct 21;17(11):e02332-26. doi: 10.1128/mbio.02332-26 (PMC13753801; doi:10.1128/mbio.02332-26)
Supplement: Supplementary file 1 [file mbio.02332-26-s0001.docx]

Supplemental Figure 1: Effect of the fungicides pyraclostrobin and metyltetraprole treatment (selection regime #2) on the frequencies of cytochrome b mutations Y132C and G143A.

Mutant Y132C (step A) was cultivated for 8 cycles with increasing concentrations of pyraclostrobin. An aliquot thereof (step B) was further treated with both metyltetraprole and pyraclostrobin for additional 2 cycles. Cell density was monitored using the Cell Growth Quantifier (CGQ). In cycle 10 and at the end of each cycle, qPCR analyses were performed. The percentages of mutations Y132C and G143A measured by qPCR are presented (steps A, B, C). At the end of cycle 10, cells were streaked onto YBG media with fungicides. After growth, single colonies were picked and further propagated (step D). Figure was created with BioRender.com.

Supplemental Figure 2: Effect of the fungicides pyraclostrobin and metyltetraprole treatment (selection regime #1) on the frequencies of cytochrome b mutations Y132C and G143A.

Mutant Y132C (step A) was cultivated for 8 cycles with increasing concentrations of pyraclostrobin. An aliquot thereof (step B) was further treated with both metyltetraprole and pyraclostrobin for additional 8 cycles. Cell density was monitored using the Cell Growth Quantifier (CGQ). At the end of each cycle, qPCR analyses were performed. The percentages of mutations Y132C and G143A measured by qPCR are presented (steps A, B, C). At the end of cycle 16, cells were streaked onto YBG media. After growth, single colonies were picked and further propagated (step D). Figure was created with BioRender.com.

Supplemental Figure 3: Multiple sequence alignment of Sanger sequenced CYTB PCR amplified gene fragments from cycle 16 population of selection regime #1.

Sequencing was done using standard M13 primer. Double mutant alleles were detected.

**Supplemental Figure 4: Multiple sequence alignment of Sanger sequenced CYTB PCR amplified gene fragments of selected UN clones.**

Sequencing was done using primer Web1973. UN109, UN113, UN114, UN116, and UN154 showed double peaks at codons 132 and 143. UN115 only displayed a GGA to GCA change at codon 143.

**Supplemental Figure 5: Multiple sequence alignment of Sanger sequenced *CYTB* PCR amplified gene fragments of MP393.**

Sequencing was done using primer Web1973, Web1974, Web2101 and Web2104. MP393 showed double peaks at codons 132 and 143.

Supplemental Figure 6: Multiple sequence alignment of Sanger sequenced CYTB PCR amplified gene fragments from MP393.

Sequencing was done using Web1973 and Web1974 primers. Double mutant alleles were detected.

**Supplemental Figure 7: Origin of analysed UN113 heteroplasmic single cells.**

Colonies #45 and #46 from UN113 grown in YBG-MP (UN113MP) were picked, resuspended and cells thereof further propagated in YBG-UN and YBG-MP. Single cells of UN113MP#45MP and UN113MP#46MP were analysed by dPCR. YBG-MP contained 0.064 µM metyltetraprole and 0.128 µM pyraclostrobin. Figure was created with BioRender.com.

**Supplemental Figure 8: Multiple sequence alignment of Sanger sequenced *CYTB* PCR amplified gene fragments of single cell MP393#4.**

Sequencing was done using standard M13 primer. Both mutant alleles were detected.
