# Supplementary material for: Mitochondrial heteroplasmy as a transitional state in the evolution of fungicide resistance
Source: mBio. 2026 Oct 21;17(11):e02332-26. doi: 10.1128/mbio.02332-26 (PMC13753801; doi:10.1128/mbio.02332-26)
Supplement: Supplementary file 2 [file mbio.02332-26-s0003.docx]

## Supplemental information. Detailed method for generation of *Saccharomyces cerevisiae* cytochrome *b* mutants by biolistic transformation

## *Saccharomyces cerevisiae* culture media and strains

The following media were used for the growth of yeast: YPD (1% yeast extract, 2% peptone, 3% glucose, 2% agar), YPG (1% yeast extract, 2% peptone, 3% glycerol, 2% agar), YPEth (1% yeast extract, 2% peptone, 2% ethanol), transformation medium (0.7% yeast nitrogen base, 3% glucose, 2% agar, 1 M sorbitol and 0.8g/l of a complete supplement mixture minus uracil, supplied by Anachem).

The following yeast strains were used in this study: W303-1B rho° (Mat α ura3 his3 leu2 ade2 trp1) devoid of mitochondrial genome, CKWT, CKGAG and CKL65 (Mat a leu1 kar1-1), and WT, G143A, Y132C and Y132C-G143A (diploid Mat α ura3 his3 leu2 ade2 trp1 / Mat a leu1 kar1-1) (**Table 1**). The strains of the CK series and the diploids have isogenic intronless mitochondrial DNA, with the exception of the point mutations in the cytochrome *b* gene *CYTB* (**Table 2**).

Table 1: List of the yeast strains and cytochrome *b* mutations

| Strain | Cytochrome *b* version | Respiratory growth competence |
| --- | --- | --- |
| CK series | | |
| CKBM | WT | wt |
| CKGAG | G143A | wt |
| CKL65 | T46K | null |
| Diploids | | |
| WT | WT | wt |
| G143A | G143A | wt |
| Y132C | Y132C | weak |
| Y132C-G143A | Y132C-G143A | null – very weak |

The strains were already available in our lab (see for instance (Fisher, Brown et al. 2004), with the exception of the double-mutant Y132C-G143A that was generated in this study.

## Generation of *S. cerevisiae* mutant strain with point mutations in the mitochondrially-encoded *CYTB* gene

The generation of the mutants were performed by the technique of biolostic transformation, using two plasmids: YEp352 which contains the *URA3* gene, allowing the selection of Ura^+^ nuclear transformants and pCytb constructed from pCRscript vector (Stratagene) and carrying the intronless version of the mitochondrial *CYTB* gene with 350 bp of the upstream sequence. In yeast, that plasmid could only be maintained in the mitochondria. Chosen mutations were introduced into the *CYTB* gene by PCR-based site-directed mutagenesis. After verification of the sequence, plasmids carrying the intended mutant *CYTB* (pCYTBmut) were used for microprojectile bombardment mediated mitochondrial transformation as previously described (see for instance (Meunier 2001). Briefly,

Microprojectile bombardment

Tungsten particles were coated with a mixture of pCYTBmut and YEp352. Aliquots of the coated particles were used for the biolistic transformation of the recipient strain W303-1B rho°. Approx. 10^9^ cells were spread evenly on transformation medium (selective for Ura^+^ transformants). The particle bombardments were performed with a Biolistic PDS 1000 He Particle Delivery System (Bio-Rad) according to the manufacturer's recommendations

Screening of transformants

Ura^+^ colonies (thus containing YEp352) appeared after 4-5 days of incubation. Amongst them, the few mitochondrial transformants (also called synthetic rho^−^) which contained the mitochondrial plasmid pCYTBmut were identified by crossing with strain CKL65, carrying the respiratory deficiency *CYTB* mutation T46K. The diploids issued form the crossing were replica-plated on respiratory medium YPG. The mitochondrial transformants (that contained pCYTBmut) were identified by their ability to form respiratory-competent diploids when crossed with CKL65, as only recombination with the plasmid derived-mitochondrial *CYTB* gene carried by pCYTBmut could correct the respiratory defect caused by T46K.

Replacement of the wt *CYTB* gene by the mutated version in a rho^+^ mitochondrial genome

The mutated *CYTB* gene was then introduced into a rho^+^ genome by double recombination, by crossing the mitochondrial transformant (or synthetic rho^−^) with a rho^+^ strain. In this study, the mitochondrial transformants identified as above were crossed with CKBM and with CKGAG, the respiratory competent strain carrying the mutation Cytb-G143A. The respiratory growth competence of approx.400 diploid colonies resulting from the crossing was tested. The respiratory deficient diploids included synthetic rho^-^ and recombinant rho^+^ carrying the double *CYTB* mutation. These latter were identified by PCR of the *COX1* gene that is encoded by the mitochondrial genome and not present in the synthetic rho^-^ (which possess only *CYTB*). The presence of the mutations was confirmed by sequencing.

Fisher, N., A. C. Brown, G. Sexton, A. Cook, J. Windass and B. Meunier (2004). "Modeling the Qo site of crop pathogens in Saccharomyces cerevisiae cytochrome b." Eur J Biochem **271**(11): 2264-2271.

Meunier, B. (2001). "Site-directed mutations in the mitochondrially encoded subunits I and III of yeast cytochrome oxidase." Biochem J **354**(Pt 2): 407-412.
